# Electronic Structure Engineering of Cu_2_O Film/ZnO Nanorods Array All-Oxide p-n Heterostructure for Enhanced Photoelectrochemical Property and Self-powered Biosensing Application

**DOI:** 10.1038/srep07882

**Published:** 2015-01-20

**Authors:** Zhuo Kang, Xiaoqin Yan, Yunfei Wang, Zhiming Bai, Yichong Liu, Zheng Zhang, Pei Lin, Xiaohui Zhang, Haoge Yuan, Xueji Zhang, Yue Zhang

**Affiliations:** 1 State Key Laboratory for Advanced Metals and Materials, School of Materials Science and Engineering, University of Science and Technology Beijing, Beijing 100083, China; 2 Research Center for Bioengineering and Sensing Technology, School of Chemistry and Biological Engineering, University of Science and Technology Beijing, Beijing 100083, China; 3 Key Laboratory of New Energy Materials and Technologies, University of Science and Technology Beijing, Beijing 100083, China

## Abstract

We have engineered the electronic structure at the interface between Cu_2_O and ZnO nanorods (NRs) array, through adjusting the carrier concentration of Cu_2_O. The electrodeposition of Cu_2_O at pH 11 acquired the highest carrier concentration, resulting in the largest interfacial electric field between Cu_2_O and ZnO, which finally led to the highest separation efficiency of photogenerated charge carriers. The optimized Cu_2_O/ZnO NRs array p-n heterostructures exhibited enhanced PEC performance, such as elevated photocurrent and photoconversion efficiency, as well as excellent sensing performance for the sensitive detection of glutathione (GSH) in PBS buffer even at applied bias of 0 V which made the device self-powered. Besides, the favorable selectivity, high reproducibility and extremely wide detection range, make such heterostructure a promising candidate for PEC biosensing applications, probably for the extended field of PEC water splitting or other solar photovoltaic beacons.

As a newly emerged technique for the detection of biomolecules, photoelectrochemical (PEC) biosensor has rapidly become a research hotspot. Due to the complete separation of excitation source (light) and detection signal (PEC current), the PEC biosensors acquire efficient reduction of some undesired background noise and improved sensitivity^1^. Nowadays, much related research is focused on the enhancement of PEC biosensor performance by incorporating numerous functional semiconductive nanomaterials. For examples, Graphene/CdSe multilayers^2^ and graphene/CdS^3^ nanocomposites were prepared for PEC analysis of thrombin and Glutathione (GSH), respectively. In addition, ZnO/graphene composite^4^, as well as ZnO/ZIF-8 nanorods architecture^5^ were employed for construction of PEC sensors. Soon afterwards, IrO2-Hemin-TiO2 nanowire arrays were also developed for PEC detection of GSH with excellent selectivity and stability^6^. More recently, bismuthoxyiodide nanoflakes/TiO2 nanotubes array based p-n heterojunction was applied in the PEC biosensing of glucose^7^. Even so, exploitation of novel semiconductor based photoanode is still urgent for advanced PEC biosensor fabrication.

The property of semiconductor heterostructure dominates the behavior of photoinduced charge carrier, which finally determines the photoelectrochemical response. As is known, a variety of Cu_2_O/ZnO p-n junctions have been applied for enhanced photocatalytic performance and efficient photovoltaic cells due to their controllable electronic structure at interface and good energy band alignment^8^,^9^,^10^,^11^,^12^,^13^,^14^,^15^,^16^,^17^,^18^,^19^. ZnO nanomaterials have been extensively studied because of their rather rich morphology world, large excitation binding energy (60 meV), deep level defects and high electron mobility^20^,^21^,^22^,^23^. However, the large band gap of ZnO (Eg = 3.37 eV) greatly restricts its utility to UV region only. Hence, Cu_2_O (Eg = 1.9 – 2.2 eV) is a promising candidate to extend the photoresponse into the visible region during PEC process^24^,^25^,^26^. Moreover, Cu_2_O is normally synthesized as a p-type semiconductor due to the copper vacancies in the lattice, and its carrier concentration is determined by the pH value of electrolyte during the electrodeposition synthesis process^27^. So this provides an approach to engineer the electronic structure at the interface of Cu_2_O/ZnO heterostructure. Besides, the non-toxicity, abundance and low cost of copper, as well as the long-term stability associated with oxides, allows for the feasibility of constructing PEC devices with environment-friendly Cu_2_O^28^,^29^.

In this work, we assembled Cu_2_O/ZnO NRs array all-oxide p-n heterostructure by directly electrodepositing p-type Cu_2_O film onto the vertically oriented n-type ZnO NRs array. As illustrated in Figure 1, the step-wise structure of energy levels constructed in the Cu_2_O/ZnO heterostructure was responsible for the mechanism of PEC biosensor. When Cu_2_O and ZnO came into contact, a p-n junction formed at their interface. There was a favorable energy band alignment for electrons transport from the conduction band (CB) of Cu_2_O to the CB of ZnO, and for holes transport from the valence band (VB) of ZnO to the VB of Cu_2_O. In the presence of illumination, the photoinduced electrons and holes were efficiently separated by the built-in interfacial electric field. The electrons transported through each individual ZnO NR and finally reached the FTO electrode to export the PEC electrical signals. While the holes migrated through Cu_2_O film and subsequently took part in the oxidation of GSH to GSSG at the surface of Cu_2_O. The quasi-1 D nature of ZnO NRs array significantly increased the interface area between ZnO and Cu_2_O, and the introduction of Cu_2_O largely improved the visible light absorption. Simultaneously, the engineered Cu_2_O/ZnO electronic structure at interface and electron transport channels of the individual ZnO NRs both contributed to the efficient separation of photoinduced charges, thus resulting in the enhanced PEC property and ideal biosensing performance for detection of GSH.

## Results

### Characterization of Cu_2_O/ZnO heterostructure

**Figure 1:**
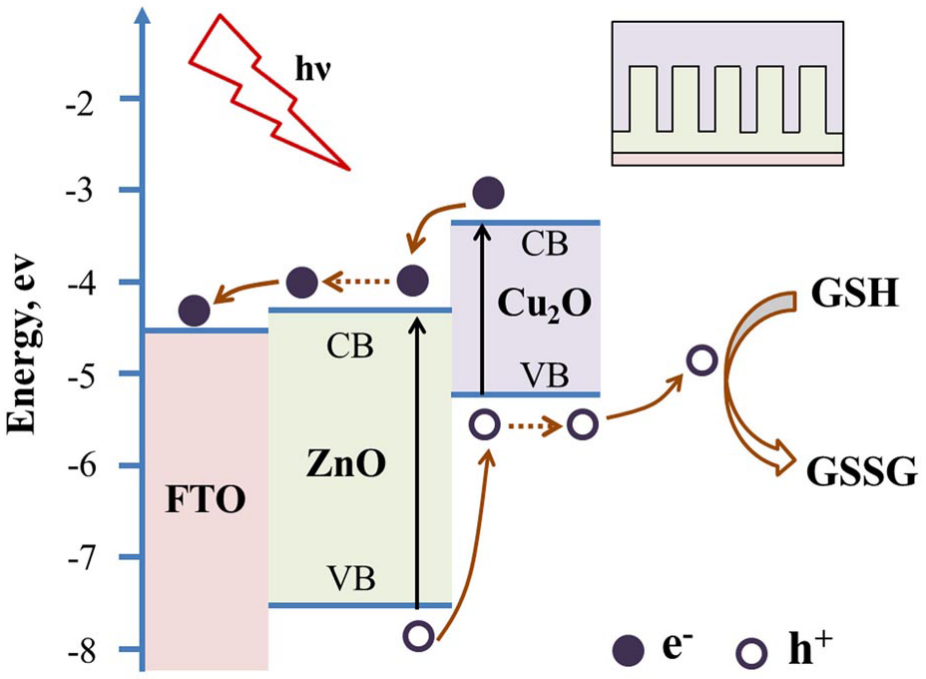
Illustration of the PEC mechanism for GSH biosensing at Cu_2_O/ZnO NRs array heterostructure based photoanode.

**Figure 2:**
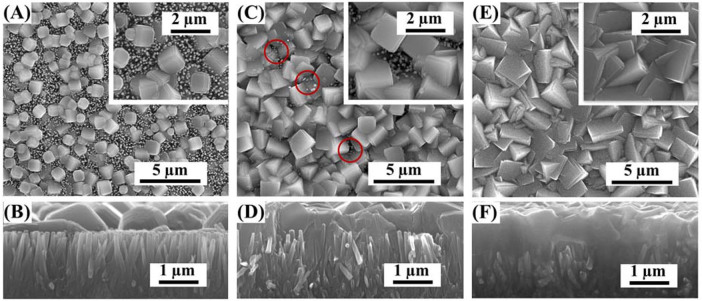
SEM images of Cu_2_O/ZnO NRs array heterostructures prepared by electrodepositon of Cu_2_O at pH 11 for 10 min (A and B), 20 min (C and D) and 30 min (E and F). The insets in (A), (C) and (E) are the close-up top views of Cu_2_O nanocrystal. (B), (D) and (F) are the side views of corresponding heterostructures.

Figure 2 shows the morphology of Cu_2_O/ZnO NRs array heterostructure prepared by electrodeposition of Cu_2_O at pH 11 for different durations. Independent ZnO NRs, with average length of ~1.8 μm and diameter of ~150 nm, were vertically grown on the FTO surface. For the electrodeposition of Cu_2_O for 10 min (Fig 1A and B), the individual Cu_2_O crystal particles spread all over the top surface of ZnO NRs array, and covered almost half of the surface area. When the electrodeposition time was increased to 20 min (Fig 1C and D), the Cu_2_O crystal particles started to form a film except for several points where ZnO was still exposed. By comparing the inset of Figure 2A and Figure 2C, the size of the Cu_2_O crystal particles obviously increased. For the samples of 30 min (Figure 2E and F), the Cu_2_O particles further expand their size, and the Cu_2_O film covered all over the ZnO NRs. By comparing Figure 2B, D and F,the thickness of Cu_2_O film increased from ~0.8 μm to ~1.1 μm along with the growth time. It is worth pointing out that the Cu_2_O crystal particles partially sank into the ZnO NRs array, so that the interface area between Cu_2_O and ZnO was significantly raised. In addition, the longer time ZnO was immersed in the alkaline electrodeposition solution, the more serious etching happened to ZnO NRs. Moreover, it is noticed that with the increase of size and amount of Cu_2_O particles, the vertical ZnO NRs suffered more stress and strain from Cu_2_O particles. However, taking all factors in terms of the PEC property into consideration, we still choose 30 min for electrodeposition of Cu_2_O film.

**Figure 3:**
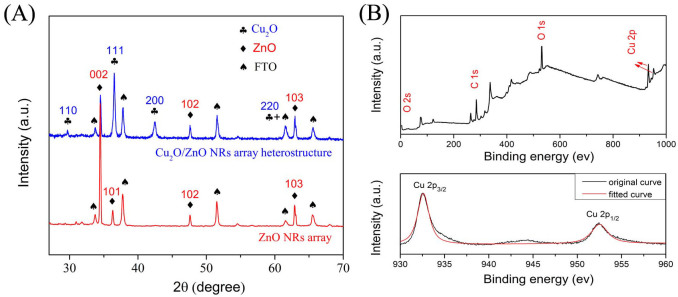
(A). XRD patterns of pristine ZnO NRs array and fabricated Cu_2_O/ZnO heterostructure at pH 11 for 30 min. (B) XPS survey spectrum of fabricated Cu_2_O/ZnO heterostructure; (C). Core level spectrum of Cu 2p in Cu_2_O.

Figure 3A shows the X-ray diffraction (XRD) patterns of the ZnO NRs array and fabricated Cu_2_O/ZnO heterostructure. The diffraction peaks of the FTO substrate were marked with spades. For pristine ZnO NRs array, diffraction peaks were identified to the hexagonal ZnO crystalline phase with a wurtzite structure. After the deposition of Cu_2_O at pH 11 for 30 min, diffraction peaks from Cu_2_O were observed in addition to those from ZnO NRs array, which can be indexed to the cubic phase Cu_2_O (JCPDS 78-2076). No diffraction peaks of impurities were found in the XRD patterns.

X-ray photoelectron spectroscopy (XPS) (Figure 3B) was performed in order to identify the chemical state of the Cu element in the heterostructure. Since XPS is only capable for surface elemental analysis, the Zn element cannot be observed in the wide scan spectrum. In the Cu 2p core level spectrum, the Cu 2p_3/2_ and Cu 2p_1/2_ spin-orbital photo-electrons were located at binding energies of 932.6 eV and 952.5 eV, respectively. Such results are in good agreement with the reported values of Cu_2_O^25^,^30^, thus demonstrating the layer electrodeposited upon ZnO NR array was Cu_2_O rather than Cu or CuO.

**Figure 4:**
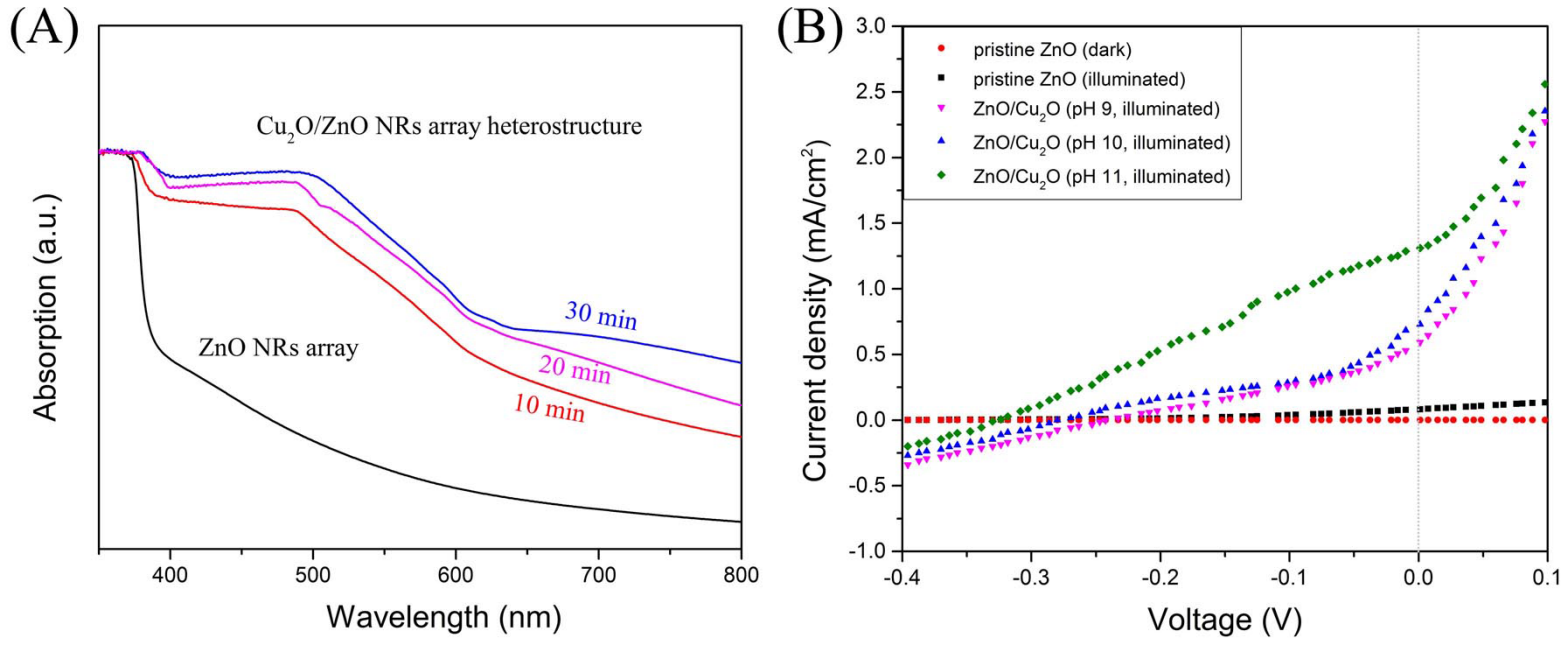
(A) UV-vis diffuse reflectance spectra of pristine ZnO NRs array and fabricated Cu_2_O/ZnO heterostructure with various electrodeposition time of Cu_2_O. (B) Linear sweep voltammagrams of pristine ZnO NRs array photoanodes without and with illumination, Cu_2_O/ZnO heterostructure electrodeposited at pH values of 9, 10 and 11 for 30 min with illumination at a scan rate of 50 mV/s under bias potentials from −0.4 to +0.1 V vs. Ag/AgCl.

Figure 4A shows the UV-vis diffuse reflectance spectra (DRS) of pristine ZnO NRs array and Cu_2_O/ZnO heterostructure with varied electrodeposition time of Cu_2_O at pH 11. The pristine ZnO resulted in an obvious absorption below 380 nm, which originated from the band edge of ZnO. And only a little absorption in the visible region can be observed, which might have resulted from the scattering effect of the NRs array structure. However, the absorption spectrum was significantly enhanced after subsequent electrodeposition of Cu_2_O. It is noteworthy that the visible light absorption difference between 10 min sample and 20 min sample was greater than that between 20 min sample and 30 min sample, which was corresponding to the isolated Cu_2_O nanocrystals for 10 min growth time, almost covering Cu_2_O film for 20 min growth time and the full covering Cu_2_O film for 30 min growth time. The strong absorption below 600 nm is attributed to the band edge absorption of the nanocrystaline Cu_2_O film. It suggests that the so-fabricated Cu_2_O/ZnO NRs array heterostructure has a quite broad absorption range from visible to ultraviolet, which well agrees with the solar irradiation. Consequently, good photoelectrochemical property of such nanostructure under white light irradiation is expected. The following PEC measurements were all based on the samples with electrodeposition of Cu_2_O for 30 min.

### Electronic structure engineering of Cu_2_O/ZnO heterostructure

**Figure 5:**
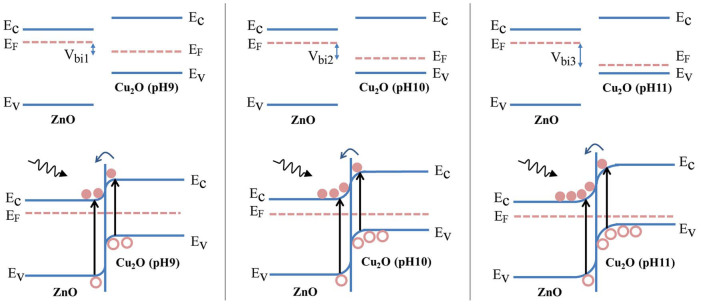
Energy band diagrams for isolated ZnO and Cu_2_O electrodeposited at various pH values from 9 to 11, and corresponding Cu_2_O/ZnO heterostructures.

Figure 4B shows the current density of pristine ZnO NRs array and Cu_2_O/ZnO heterostructures electrodepostied at various pH values without and with illumination as the function of applied bias potential. For pristine ZnO, the dark current density is on the order of 10^−3^ mA/cm^2^, and the photocurrent density has a slight increasment. While all samlpes with Cu_2_O/ZnO heterostructure showed pronounced photoresponse under illumination, which can be attributed to the improved visible light absorption and efficient interfacial charge transport of Cu_2_O/ZnO heterostructure. Noticeably, the photoresponse was gradually improved along with the increase of the electrodepostion pH value from 9 to 11. The saturated photocurrent density of Cu_2_O (pH 11)/ZnO was approximately 13.5 times that of pristine ZnO NRs array, 2.3 times that of Cu_2_O (pH 9)/ZnO, and 1.7 times that of Cu_2_O (pH 10)/ZnO at 0 V bias. Such results can be explained by the different carrier concentrations of Cu_2_O in the samples, which is demostrated by Mott-Schottky plots (Figure S2). During the liquid electrodeposition of Cu_2_O, the carrier concentration of Cu_2_O was adjusted by changing the pH value of precusor solutions^31^,^32^, which in turn led to the alteration of the built-in potential in Cu_2_O/ZnO hetrostructure^14^,^15^. As illustrated in Figure 5, the built-in potential V_bi_ is equal to the difference between the Fermi levels of ZnO and Cu_2_O. Consequently, the samples with higher electrodepostion pH value, that is with higher carrier oncentration of Cu_2_O, possess larger built-in potential. When the illumination was applied, the photoinduced electron-hole pairs were separated under the effect of the interfacial electric field. Therefore, Cu_2_O (pH 11)/ZnO got the highest charge separation efficiency, which resulted in the best photoresponse at bias potential of 0V.

Based on the discussion above, Cu_2_O/ZnO heterostructure electrodeposited at pH value of 12 or 13 should perform better photoresponse. However, in such strong alkaline electrodepostion electrolytes, the dissolution and etching of ZnO NRs array is very serious, which would certainly introduce a high concentration of interface states at the Cu_2_O/ZnO heterostructure, thus to a large extent affecting the quality of the heterostructure^10^,^14^. Moreover, considering the stability of the fabricated devices, pH 11 is the optimal parameter for construction of Cu_2_O/ZnOheterostructure based PEC biosesnsor with ideal performance. So the following PEC measurements were based on the samples acquired at pH 11.

### PEC property of Cu_2_O/ZnO heterostructure photoanode

**Figure 6:**
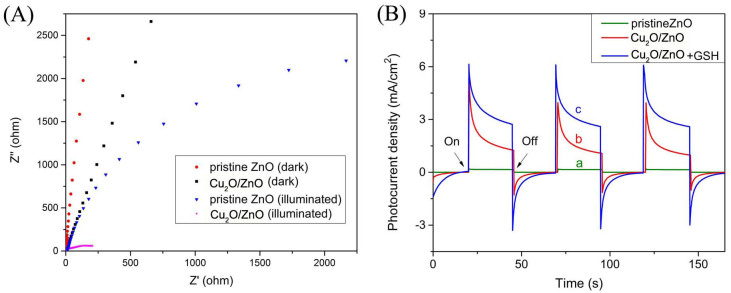
(A) EIS Nyquist plts of pristine ZnO NRs array and Cu_2_O/ZnO hetrostructrue with or without illumination in 0.1 M PBS at bias potential of 0 V vs.Ag/AgCl. (B) Photoresponse of pristine ZnO NRs array (a) and Cu_2_O/ZnO heterostructure (b) in 0.1 M PBS, as well as Cu_2_O/ZnO heterostructure in 0.1M PBS containing 200 μM GSH (c) under illumination at bias potential of 0 V vs. Ag/AgCl.

Electrochemical impedence spectroscopy (EIS) was carried out both in the dark and under illumination at bias potential of 0 V vs. Ag/AgCl. It is known that the semicircle in the Nyquist plot at high frequencies is the characteristic of the charge transfer process, and the diameter of the semicircle is equal to the charge transfer resistance (Rct). As shown in Figure 6A, the Rct of Cu_2_O/ZnO heterostructure was apparently much smaller than that of the pristine ZnO NRs array under illumination. Such result indicates that the formation of Cu_2_O/ZnO heterostructure dramatically promoted the interfacial charge transport and the separation efficiency of photoinduced charges under illumination, thus demonstrating the enhanced PEC performance for Cu_2_O/ZnO NRs array heterostructure.

PEC behaviors of pristine ZnO NRs array and Cu_2_O/ZnO heterostructure with and without addition of GSH at bias potential of 0V were also characterized in Figure 6B. No photocurrent was observed in the dark. Upon photoexcitation, the photocurrent composed of three steps: (a) the photocurrent climbed promptly; (b) the photocurrent decreased sharply; (c) the photocurrent gradually reached a stable state. These three stages are corresponding with the photoinduced electrons under illumination, the recombination of photoinduced electron-hole pairs, and the balance of generation and recombination process, respectively^25^. The pristine ZnO NRs array photoanode performed an average photocurrent density of 0.097 mA/cm^2^ (curve a), whereas the Cu_2_O/ZnO heterostructure showed an average photocurrent density of 1.35 mA/cm^2^ (curve b), demonstrating the remarkable improvement of photoelectric conversion efficiency. The formed p-n heterojunction between Cu_2_O and ZnO provided an interfacial electric field, which subsequently contributed to the charge separation efficiency, and finally led to the significantly enhanced photoresponse. Moreover, the elevated photocurrent could be further strengthened with the presence of sacrificial reagent who served as the hole scavenger. Since GSH plays an important role in many biological functions, it was adopted as a model molecule to demonstrate the feasibility of fabricating Cu_2_O/ZnO heterostructure based PEC biosensor. When GSH was added in the blank PBS (200 μM), the average photocurrent density reached 2.91 mA/cm^2^ (curve c). The great photocurrent change indicated the fabricated Cu_2_O/ZnO heterostructure is suitable for detecting GSH based on PEC method.

There is no doubt that when a positive bias potential is applied, the photocurrent density will apparently increase due to the more efficient charge separation and longer lifetime of photoinduced electron-hole pairs. Neverthless, in order to realize the self-powered function, plus taking energy conservation and the selectivity of PEC biosensors into consideration, 0 V was selected as bias potential in this work.

### Self-powered PEC biosensing of GSH

**Figure 7:**
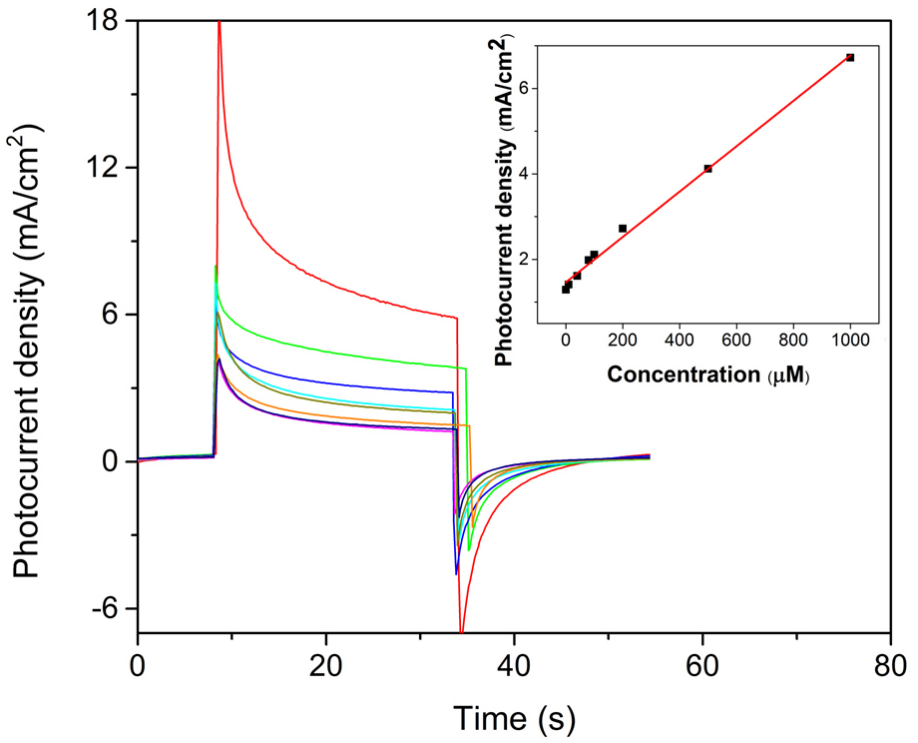
Photocurrent responses of Cu_2_O/ZnO heterostructure based photoanode in 0.1 M PBS in the presence of 0, 10, 40, 80, 100, 200, 500, 1000 μM GSH (from bottom to top) at 0 V (vs. Ag/AgCl) under illumination. Inset: linear calibration curve.

In order to further verify the sensitive sensing of GSH based on Cu_2_O/ZnO heterostructure, Figure 7 displays the photocurrent response in the presence of GSH with various concentrations at applied bias potential of 0 V (vs. Ag/AgCl). The photocurrent density performed a fine linear relationship with the concentration of GSH from10 to 1000 μM (R^2^ = 0.991), which is much wider than that of porous TiO_2_-Pt nanowhisker^33^, flower-like Cu_2_O/ZnO^29^ and IrO_2_-Hemin-TiO_2_^6^ photoanodes based on PEC method, as well as Au nanoparticles@Si nanowires^34^, Hg/Pd^35^ and ordered mesoporous carbon^36^ photoanodes based on electrochemical method. The detection limit was estimated as low as 0.42 μM through 3σ. The detailed performance comparisons with previously reported literatures are listed in Table S1. Obviously, the proposed Cu_2_O/ZnO NRs array heterostructure based PEC biosensor shows great promise for applications in the monitoring of GSH with high sensitivity and wide linear range.

**Figure 8:**
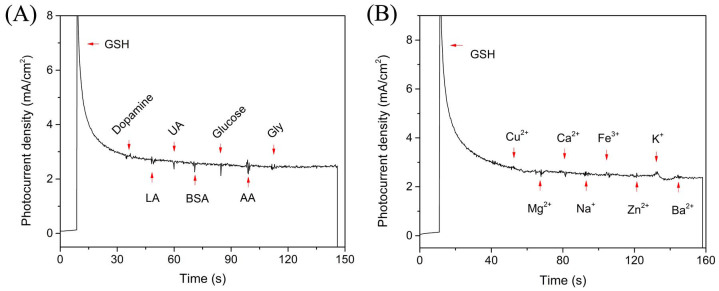
Photocurrent responses of Cu_2_O/ZnO heterostructure based PEC biosensors in the presence of GSH (200 μM) and (A) dopamine, LA, UA, BSA, glucose, AA, Gly with the concentration of 50 μM, as well as (B) various metal ions such as Cu^2+^, Mg^2+^, Ca^2+^, Na^+^, Fe^3+^, Zn^2+^, K^+^ and Ba^2+^ with the concentration of 50 μM at bias potential of 0 V (vs. Ag/AgCl).

The selectivity of Cu_2_O/ZnO heterostructure based PEC biosensor was confirmed by measuring the photocurrent response not only for common chemical or biological interferences but also for various metal ions with the concentration of 50 μM, showing much smaller or negligible signals compared to that of GSH with the concentration of 200 μM (Figure 8A and B). The excellent selectivity is ascribed to the low applied bias potential which minimized the interference from other reductive species^6^,^37^. Furthermore, the relative standard deviation (R.S.D) of the photoresponse to 200 μM GSH was 5.2% for six successive measurements. The R.S.D for detection of 200 μM GSH with six different Cu_2_O/ZnO based photoanode under the same condition was 6.4%. In addition, the photocurrent density was stable for 2 h in 0.1 M PBS containing 10 μM GSH (Figure S5), and 91% of the initial photocurrent response was maintained after storing for over half month in the repeating test (Figure S6). All the results demonstrated the reproducibility and stability of fabricated heterostructures and their practical potential for PEC biosensing application.

## Discussion

The enhanced PEC property and excellent PEC biosensing performance was probably attributed to the following five points: (a) The quasi 1-D nature of ZnO NRs array offered large p-n heterojunction interface area when in combination with Cu_2_O film; (b) The introduction of Cu_2_O significantly improved the visible light absorption, so that the solar energy could be utilized more sufficiently; (c) The electronic structure between Cu_2_O and ZnO was engineered thorough adjusting the carrier concentration in Cu_2_O and the thickness of Cu_2_O film. Thereby, the optimal build-in potential was acquired at the Cu_2_O/ZnO interface, resulting in the efficient charge separation; (d) The step-wise energy band structure facilitated the photoinduced electrons to the electrode while promoted the photoinduced holes to accumulate in the VB of Cu_2_O and subsequently to be consumed by participating in the oxidation of GSH. This process further prevented the recombination of photogenerated carriers; (e) The individual and vertical alignment of single crystal ZnO NRs provides transport channels for electrons to rapidly reach FTO electrode.

To conclude, the Cu_2_O/ZnO NRs array all-oxide p-n heterostructure was successfully fabricated, and the electronic structure at the interface between Cu_2_O and ZnO was engineered through adjusting the carrier concentration of Cu_2_O. The results demonstrated the electrodeposition of Cu_2_O at pH 11 acquired the highest carrier concentration, resulting in the largest build-in electric field, which finally led to the highest separation efficiency of photoinduced charge carriers. The optimized heterostructure exhibited enhanced PEC performance and was subsequently adopted for self-powered PEC sensing of GSH. The Cu_2_O/ZnO based PEC biosensor performed a linear range from 10 μM to 1000 μM and an estimated detection limit of 0.42 μM even at bias potential of 0 V. Moreover, the excellent selectivity, reproducibility and stability suggested that such heterostructure is a competitive candidate for advanced PEC biomolecular detection, maybe for the extended field of PEC water splitting or other solar photovoltaic beacons.

## Methods

### Reagents

Glutathione (GSH), ascorbic acid (AA), uric acid (UA), lactic acid (LA), glucose, dopamine, bovine serum albumin (BSA), Glycine (Gly), polyetherimide (PEI) were purchased from Sigma-Aldrich. 0.1 M pH 7.4 phosphate buffer saline (PBS) was always employed as the supporting electrolyte. Zinc acetate (Zn(CH_3_COO)_2_.2H_2_O), Zinc nitrate hexahydrate (Zn(NO_3_)_2_.6H_2_O], hexamethylenetetramine (HMTA, (CH_2_)_6_N_4_), Copper sulfate (Cu_2_SO_4_) and sodium hydroxide (NaOH) were purchased from Beijing chemical reagent company. Other reagents were of analytical grade and all aqueous solutions were prepared with deionized water.

### Apparatus

The samples were characterized by Field emission scanning electron microcopy (FESEM, Zeiss, SUPRA-55, German), X-ray diffractometer (XRD, Rigaku DMAX-RB, Japan, CuKα), X-ray photo-electron spectroscopy (XPS, Axis UltraDLD, SHIMADZU, Japan).The PEC related experiments were carried out using an electrochemical workstation (Solartron SI1287/SI 1260) under AM 1.5G illumination provided by a solar simulator (Oriel, 91159A, 100 mW/cm^2^).

### Preparation of Cu_2_O/ZnO NRs array based photoanode

The ordered ZnO NRs array was prepared by the hydrothermal growth method on fluorine doped tin oxide (FTO) substrates^38^,^39^,^40^. The ZnO seed layer was acquired by spin coating a colloidal solution of Zn(CH_3_COO)_2_.2H_2_O (0.5 M) onto the FTO substrate, and subsequently annealed at 350 °C in air for 30 min. In the hydrothermal process, Zn(NO_3_)_2_.6H_2_O and HMTA were dissolved in deionized (DI) water with equal concentration of 50 mM. Besides, PEI (5 mM) was dissolved in the solution to enhance the aspect ratio of the NRs array. The substrate coated with the ZnO seed layer was placed in the precursor solution at 90 °C for 3 h, and then rinsed with DI water for three times.

The electrodeposition of Cu_2_O thin film was conducted with a three-electrode system in water bath (40 °C). Aqueous solution containing Cu_2_SO_4_ (0.05 M) and LA (0.1 M) was prepared, and the pH value was adjusted to 9.0, 10.0, 11.0 and 12.0 through dropwise adding NaOH (4 M). Afterward, the prepared ZnO NRs array was immersed into the resulting solution under the potential of −0.4 V vs. Ag/AgCl for 10 min, 20 min and 30 min. Finally, the prepared Cu_2_O/ZnO heterostructure was again rinsed with DI water for three times and dried in nitrogen gas.

## Author Contributions

Y.Z. and Z.K. designed the experiments and wrote the manuscript text. Y.L., Xi.Z. and H.Y. carried out the material synthesis and characterization. Z.K., Y.W. and X.Y. analyzed the data. Z.B., Z.Z., P.L., and Xu. Z. provided the support for theoretical explanation. All authors discussed the results and commented on the manuscript.

## Supplementary Material

Supplementary Information Supplementary Information
