# Supplementary material for: Electronic Structure Engineering of Cu2O Film/ZnO Nanorods Array All-Oxide p-n Heterostructure for Enhanced Photoelectrochemical Property and Self-powered Biosensing Application
Source: Sci Rep. 2015 Jan 20;5:7882. doi: 10.1038/srep07882 (PMC4298735; doi:10.1038/srep07882)
Supplement: Supplementary Information [file srep07882-s1.pdf]

Electronic Structure Engineering of Cu_2O Film/ZnO Nanorods Array All-Oxide p-n Heterostructure for Enhanced Photoelectrochemical Property and Self-powered Biosensing Application

Zhuo Kang,[1] Xiaoqin Yan,[1] Yunfei Wang,[1] Zhiming Bai,[1] Yichong Liu,[1] Zheng Zhang,[1] Pei Lin,[1] Xiaohui Zhang,[1] Haoge Yuan,[1] Xueji Zhang,[2] Yue Zhang[1,3,*]

[1]State Key Laboratory for Advanced Metals and Materials, School of Materials Science and Engineering, University of Science and Technology Beijing, Beijing 100083, China, [2]Research Center for Bioengineering and Sensing Technology, School of Chemistry and Biological Engineering, University of Science and Technology Beijing, Beijing 100083, China, [3]Key Laboratory of New Energy Materials and Technologies, University of Science and Technology Beijing, Beijing 100083, China.

*Email: yuezhang@ustb.edu.cn

Introduction of glutathione

Glutathione (GSH) is a tripeptide composed of glutamyl (Glu), cysteine (Cys), and glycine (Gly) and endogenous antioxidant, which distributed widely in the intracellular environment[1, 2]. GSH is mainly found playing a direct or indirect role in gene expression regulation, enzyme activity and metabolic regulation, cell protection, amino acid transport, immune regulation, etc. Therefore, the level of GSH is associated with many diseases including rheumatoid arthritis, muscular dystrophy, amyotrophic lateral sclerosis, Alzheimer's disease and Werner syndrome[3-7]. Upon oxidation, GSH is transformed to glutathione disulfide (GSSG), also called L-(-)-glutathione. The detailed molecular formulas are shown in **Figure S1**.

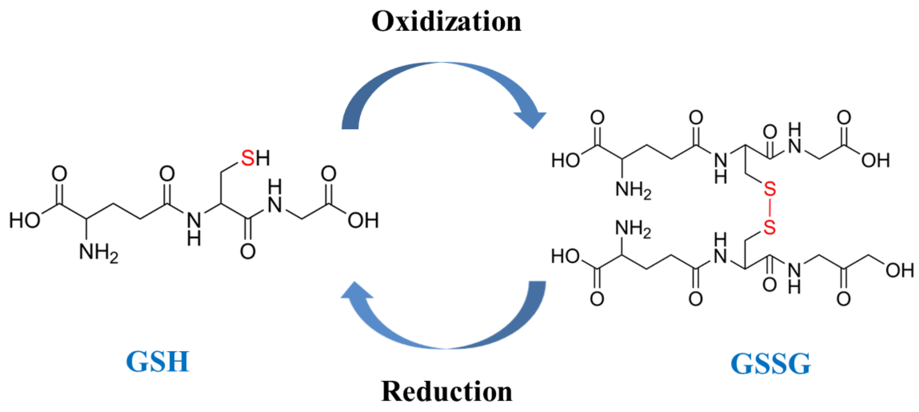

Figure S1 | The molecular formulas of GSH and GSSG.

The carrier concentration difference of Cu_2O samples

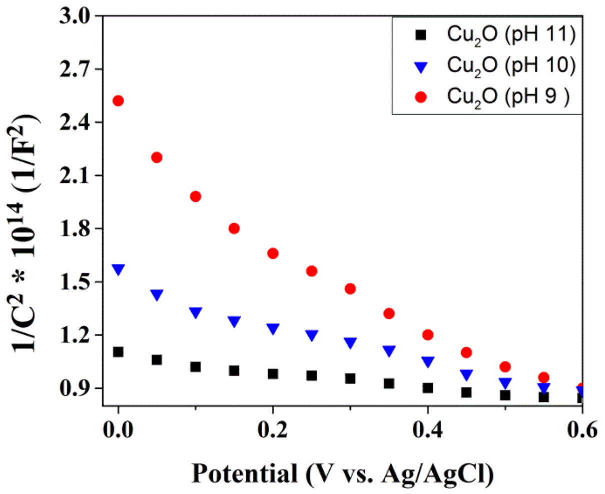

Figure S2 | Mott-Schottky plots of the Cu_2O electrodeposited at different pH values in the dark at frequencies of 5 kHz with a three-electrode system.

Cu_2O samples electrodeposited on FTO at various electrodeposition pH were characterized by electrochemical impedance measurements. The potential sweep was performed in the dark at a fixed frequency (5 KHz) with alternating current (AC) amplitude of 50 mV. The negative slopes in the **Figure S2** demonstrate that all the Cu_2O samples acquired at various pH values are p-type semiconductors. And the acceptor concentration in Cu_2O can be estimated according to the Mott-Schottky equation:

$$1/C^2 = 2/e\varepsilon\varepsilon_0 A^2 N_A (V-V_{FB} + kT/e) \qquad [1]$$

where C represents the capacitance of the space charge region, e is the electronic charge, ε is the relative permittivity of Cu_2O ($\varepsilon \sim 6.3$), ε_0 is the permittivity of vacuum, A is the area of the working electrode, V is the applied potential, V_{FB} is the flatband potential, k is Boltzmann's constant, T is the absolute temperature, and N_A is the carrier density. The carrier concentration were calculated to be 2.56×10^{16} cm^{-3}, 9.34×10^{16} cm^{-3} and 3.95×10^{17} cm^{-3} for Cu_2O (pH 9), Cu_2O (pH 10) and Cu_2O (pH 11), respectively. These data are comparable to the reported values of Cu_2O[8, 9]. Therefore, the carrier concentration of Cu_2O increases with the electrodeposition pH.

The stability of Cu_2O/ZnO photoanodes

In order to characterize whether the photo-corrosion of Cu_2O took place during the PEC biosensing measurement, XPS spectra was again performed after the tests (**Figure S3**). The Cu $2p_{3/2}$ and Cu $2p_{1/2}$ spin-orbital photo-electrons were located at binding energies of 932.6 eV and 952.5 eV, respectively. It is still in good agreement with the reported values of Cu_2O rather than CuO or Cu. Hence, the results demonstrated that the Cu_2O film electrodeposited on the ZnO NRs array is rather stable.

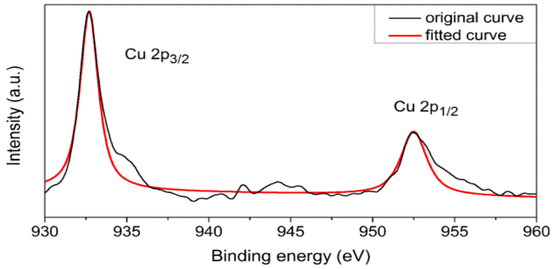

Figure S3 | The XPS spectrum of Cu 2P for Cu_2O/ZnO heterostructure after PEC measurement.

Figure S3 shows the SEM image of surface morphology of Cu_2O/ZnO heterostructure after PEC measurements. It can be clearly seen that Cu_2O crystal particles remained their original morphology, except for some stain spots left on Cu_2O surface which might be the residual of PEC electrolyte after dried in the air. This again confirmed the stability of the fabricated photoanodes.

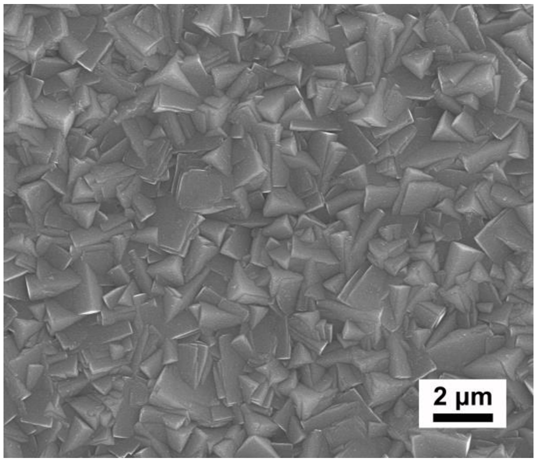

Figure S4 | SEM image of the surface morphology of Cu_2O/ZnO heterostructure after PEC measurements.

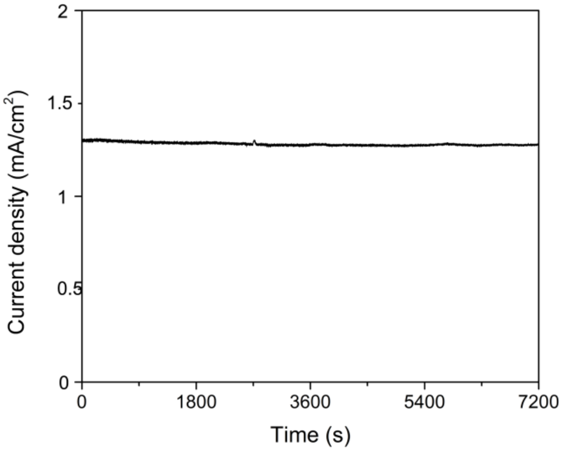

Figure S5 | Time-dependent photocurrent density of the Cu_2O/ZnO heterostructure based photoanode under continuous illumination in 0.1 M PBS containing 10 µM GSH.

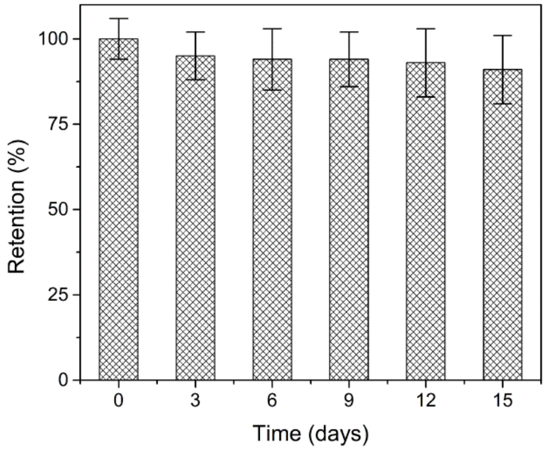

Figure S6 | The stability test of Cu_2O/ZnO heterostructure based PEC biosensor.

Table S1 Analytical performance of the ZnO/Cu_2O based photoanode for detection of GSH compared with previously reported literatures

Methods	Materials	LR (μM)	LOD (μM)	Reference
**PEC**	Cu_2O/ZnO NRs array	10-1000	0.42	This work
**PEC**	Porous TiO_2-Pt	0.5-40	0.1	[7]
**PEC**	Flower-like Cu_2O/ZnO	1-10, 20-100	0.8	[10]
**PEC**	Graphene-CdS	10-1500	3	[11]
**PEC**	IrO_2-Hemin-TiO_2	0.01-10	0.01	[12]
**EC**	Au NPs@Si NWs	0.33-2.97	0.33	[13]
**EC**	Hg/Pd	25-150	8.1	[14]
**EC**	$Cu(OH)_2$-carbon ionic liquid electrode	1-50	0.03	[15]
**EC**	Ordered mesoporous carbon	3-130	0.1	[16]
**ECL**	CdTe QD-graphene oxide	24-214	8.3	[17]

LOD = limit of detection; LR = linear ranges; ECL = electrochemiluminescence; NP = nanoparticle; NW = nanowire

Reference

1. Gutscher, M. *et al.* Real-time imaging of the intracellular glutathione redox potential. *Nature methods* **5**, 553-559 (2008).
2. Deng, R. *et al.* Intracellular glutathione detection using MnO_2-nanosheet-modified upconversion nanoparticles. J. Am. Chem. Soc. 133, 20168-20171 (2011).
3. Gambhir, J. K., Lali, P. & Jain, A. K. Correlation between blood antioxidant levels and lipid peroxidation in rheumatoid arthritis. *Clin. Biochem.* **30**, 351-355 (1997).
4. Samiec, P. S. *et al.* Glutathione in human plasma: decline in association with aging, age-related macular degeneration, and diabetes. *Free Radical Bio. Med.* **24**, 699-704 (1998).
5. Bonnefont-Rousselot, D. Blood oxidative stress in amyotrophic lateral sclerosis. *J. Neurol. Sci.* **178**, 57-62 (2000).
6. Cecchi, C. *et al.* Gluthatione level is altered in lymphoblasts from patients with familial Alzheimer's disease. *Neurosci. Lett.* **275**, 152-154 (1999).
7. Chen, G. *et al.* Photoelectrocatalytic oxidation of glutathione based on porous TiO_2–Pt nanowhiskers. *Langmuir* **28**, 12393-12399 (2012).
8. Felipe, C. B. *et al.* Direct observation of the valence band edge by in situ ECSTM-ECTS in p-type Cu_2O layers prepared by copper anodization. *J. Phys. Chem. C*, **113**, 1028–1036 (2009).
9. Jiang, T. *et al.* Carrier concentration-dependent electron transfer in Cu_2O/ZnO nanorod arrays and their photocatalytic performance. *Nanoscale* **5**, 2938-2944 (2013).
10. Li, J. *et al.* Facile electrodeposition of environment-friendly Cu_2O/ZnO heterojunction for robust photoelectrochemical biosensing. *Sens. Actuators, B* **191**, 619-624 (2014).
11. Zhao, X. *et al.* Fabrication of glutathione photoelectrochemical biosensor using graphene-CdS nanocomposites. *Analyst* **137**, 3697-3703 (2012).
12. Tang, J. et al. Photoelectrochemical detection of glutathione by IrO_2-Hemin-TiO_2 nanowire arrays. *Nano Lett.* **13**, 5350-5354 (2013).
13. Yang, K. et al. Gold nanoparticle modified silicon nanowires as biosensors. N*anotechnology* **17**, S276 (2006).
14 Antwi, C. *et al.* Use of microchip electrophoresis and a palladium/mercury amalgam electrode for the separation and detection of thiols. *Anal. Methods* **3**, 1072-1078 (2011).
15. Safavi, A. *et al.* Simultaneous electrochemical determination of glutathione and glutathione disulfide at a nanoscale copper hydroxide composite carbon ionic liquid electrode. *Anal. Chem.* **81**, 7538-7543 (2009).
16. Ndamanisha, J. C. *et al.* Application of electrochemical properties of ordered mesoporous carbon to the determination of glutathione and cysteine. *Anal. Biochem.* **386**, 79-84 (2009).
17. Wang, Y. *et al.* Graphene oxide amplified electrogenerated chemiluminescence of quantum dots and its selective sensing for glutathione from thiol-containing compounds. *Anal. Chem.* **81**, 9710-9715 (2009).
